# Early and extraordinary peaks in physical performance come with a longevity cost

**DOI:** 10.18632/aging.101023

**Published:** 2016-08-19

**Authors:** Paul L van de Vijver, David van Bodegom, Rudi GJ Westendorp

**Affiliations:** ^1^ Leyden Academy on Vitality and Ageing, 2333 AA Leiden, the Netherlands; ^2^ Leiden University Medical Center, 2333 ZA Leiden, the Netherlands; ^3^ Department of Public Health and Center for Healthy Aging, University of Copenhagen, 1165 Copenhagen, Denmark

**Keywords:** life history theory, athletes, personal record, trade-off, development, longevity

## Abstract

Life history theory postulates a trade-off between development and maintenance. This trade-off is observed when comparing life histories of different animal species. In humans, however, it is debated if variation in longevity is explained by differences in developmental traits. Observational studies found a trade-off between early and high fecundity and longevity in women. Development encompasses more than fecundity and also concerns growth and physical performance. Here, we show a life history trade-off between early and above average physical performance and longevity in male Olympic athletes. Athletes who peaked at an earlier age showed 17-percent increased mortality rates (95% CI 8-26% per SD, p<0.001) and athletes who ranked higher showed 11-percent increased mortality rates (95% CI 1-22% per SD, p=0.025). Male athletes who had both an early and extraordinary peak performance suffered a 4.7-year longevity cost. (95% CI 2.1-7.5 years, p=0.001). This is the first time a life history trade-off between physical performance and longevity has been found in humans. This finding deepens our understanding of early developmental influences on the variation of longevity in humans.

## INTRODUCTION

Generalized life history theory postulates a trade-off between development and maintenance explaining the considerable variation of traits like age at maturation, age at first reproductive event, number of offspring, size and lifespan across and within species [1-3]. It is debated whether the variation in human lifespan can also be explained by such trade-offs. Observational studies in women have shown early and above average fecundity to come at a cost of longevity [4-8]. The life history of men has no distinct mark of the end of development as menarche in women, but negative correlations between number of offspring and life-span after age 50 have also been reported for males [7, 8]. This could be explained as males invest more in physical strength and growth, which is associated with attractiveness and dominance, two traits important for male fitness [9, 10].

Professional athletes push their physical performance to the maximum and keep accurate track of these achievements. Consequently, their personal record is an accurate representation of the age of their peak performance. Under the assumption that professional athletes train at maximum intensity, this peak performance is an accurate read-out of the maximal physiological capacity of the individual. Because athletes compete intensely, the rank of peak performances is an accurate comparison of these maximal physiological capacities of athletes. According to theory of life history regulation, the period before the peak performance could be considered as development, while the decline in physical capabilities after setting a personal record is a hallmark of the ageing process [11, 12].

## RESULTS

We used a unique historical cohort of 1055 Olympic track and field athletes from 41 different nationalities from the Olympic Games from 1896 through 1936 [13]. Track and field is a large group of similar sports for individual performance where the results are measured on a continuous scale. Technological advancements contribute only little to basic body functions like running, throwing and jumping, which are critically dependent on physical strength and coordination. Athletic games are therefore an ideal group of sports to use in this study. Of these Olympic athletes 958 were men and 97 women, competing in 58 disciplines. Most historical athletes competed in several disciplines and therefore we had information on 2320 personal records. Mean age at personal record was 24.9 years (SD 3.8). The year of birth ranged between 1864 and 1913, and the year of death ranged between 1901 and 2010. Mean age at death was 72.1 years (SD 16.9). (See Supplementary Information).

To compare peak performance of athletes from different disciplines and sexes we standardized age at, and rank of the personal record per discipline and sex. Athletes who had a peak performance one standard deviation earlier showed 17-percent increased mortality rates compared to those who reached their personal record later in life (95% CI 8-26%, P<0.001). Independent of the age of their personal record, athletes who ranked one standard deviation higher than their peers showed 11-percent increased mortality rates compared to those who were ranked lower (95% CI 1-22%, p=0.025).

**Figure 1:**
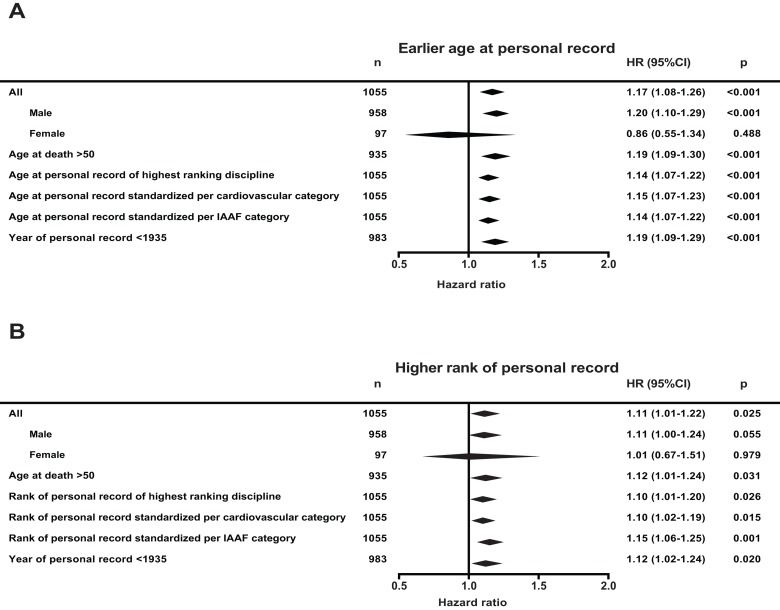
Hazard ratio (HR) for mortality of all Olympic athletes under study and several additional analyses (**A**) Hazard ratio per standard deviation younger age at personal record and (**B**) per standard deviation higher of rank of personal record. Hazard ratios were derived from a multivariate left truncated Cox' regression model, adjusted for nationality, sex, year of birth and respectively rank of personal record or age at personal record. Main analysis is with all 1055 Olympic athletes. ‘Age at death >50’ indicates that all athletes who died before age 50 were excluded. For the ‘Age of personal record of the highest ranking discipline’ analysis, we used an athlete's relative best discipline to calculate age and rank of peak performance. In the analyses ‘Standardized per cardiovascular category and IAAF category’, we grouped and standardized age and rank of personal record per cardiovascular intensity or per IAAF category (see methods). In the ‘Year of personal record <1935’ analysis, all personal records set after the year 1935 were excluded.

Figure 1 presents outcomes of various additional analyses. First, we analyzed males and females separately. Because of the small number of women in the early Olympic Games under study here, the risk estimates for women have very wide confidence intervals, but were not significantly different from men. Second, to exclude the influence of accidental causes of mortality directly related to professional sports, we analyzed survival from age 50 onwards. The outcomes did not differ. Third, instead of compiling their multiple personal records, we only analyzed the personal record in the athlete's primary discipline, the highest ranking discipline, which provided similar outcomes. Fourth, we performed analyses in which we standardized the personal records not per Olympic discipline but per cardiovascular intensity category as classified by the American college of cardiology, and per category as classified by the IAAF as we have done earlier [14-16]. The estimates were unaffected. Finally, we considered whether the outcomes could have been influenced by any kind of performance enhancing drugs. The first doping that was proven effective was developed in 1935; we therefore assumed that before that year an effect of doping was excluded [17, 18]. Using the 92% of the observations that were achieved before 1935 yielded similar results (Figure 1). It is important to note that cocaine was available since the first Olympic Games and could have played a role in the association.

**Figure 2:**
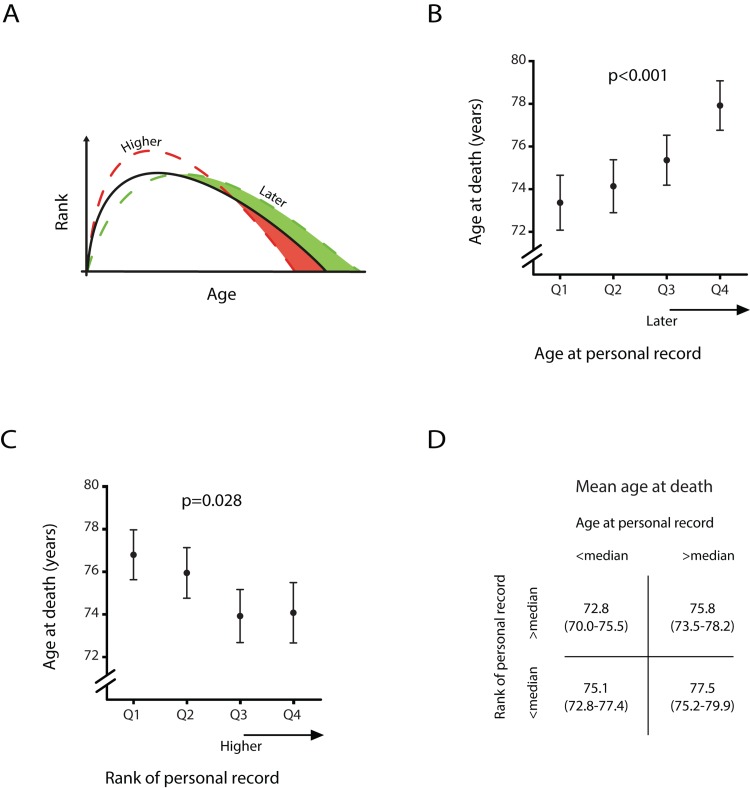
Age at death of male athletes dependent on the age and rank of their personal record (**A**) Predicted scenarios by life history theory with a later age at personal record and a higher performance score of personal record. (**B**) Estimated mean age at death per quartile of age at personal record and (**C**) estimated mean age at death per quartile of performance score of personal record. Error bars denote one standard error. (**D**) Estimated mean age at death and between brackets the corresponding 95% confidence interval shown for four different groups. All estimates are derived from a linear mixed model with only males who died after age 50, adjusted for sex, year of birth, nationality and respectively rank of personal record or age at personal record.

Life history regulation presumes a compromise between variations in the age at, and the extent of physical development and longevity (see Figure 2A) [19]. Figure 2 also presents the average age at death depending on the age and the rank of the personal record. Since hazard ratios of mortality for women were not significant associated with age or rank of personal record, the following estimates were calculated in males only. Male athlete's age at death increased from 73.4 (SE 1.3) years in those who had their peak performance in the 25% youngest age range to 77.9 (SE 1.2) years in athletes who reached their personal record in the 25% oldest age range (panel B, p<0.001). Panel C shows the association between the rank of the personal record and the age at death. The age at death decreased from 76.8 (SE 1.7) years in those who had their peak performance in the 25% lowest ranks to 74.1 (SE 1.4) years in male athletes who reached their personal record in the 25% highest ranks (p=0.028). Panel D shows the joint effect of age at, and rank of the personal record. We found that the mean age at death was lowest (72.8, SE 1.4) for those male athletes whose personal record was in the earlier half of age and better half of rank. Male athletes who peaked at a later age and had a relatively minor rank died at a mean age of 77.5 (SE 1.2), resulting in a 4.7 (SE 1.4) year difference in life expectancy between the two groups (p=0.001).

## DISCUSSION

Several factors have to be considered when interpreting the present findings. First, differences in socioeconomic status could have distorted the observations as wealthier athletes may have had better training and therefore gained better personal records at an earlier age. An affluent environment during development is not only likely to contribute to earlier and better physical performance, wealthy people on average live longer than poor people. We did not have information on the socioeconomic position of the athletes, but differences are unlikely to explain for the observed trade-off, for they would have worked in the opposite direction and, if anything, they may have masked the associations and any true biological relation would even be stronger. We also had no information on the height of athletes.

Early cessation of training and competition of individual athletes could have resulted in an underestimation of the age, and the rank of the personal record. Personal records however, have been recorded not only at the Olympic Games but also at every other official competition, and we assumed that all athletes were determined to maximize their performance over a prolonged period and would only stop prematurely in rare instances. Even if early cessation was frequent, it would have weakened the observed association between peak performance and age at death and again, any true relation would be stronger than we observed here.

According to expectation, we observed a gradual increase in life expectancy of athletes from later birth cohorts (data not shown). Moreover, a recent year of birth was also related to a better personal record at an earlier age. This ongoing professionalization of sports thus masked the observed trade-off; we therefore adjusted for birth cohort.

It is difficult to interpret the outcomes among women. One possibility is that there is a sex dimorphism and the effect is smaller in women, but we were not able to study this properly because of the relatively small number of female athletes. The alternative is that the variance is greater but the effect size is not different. It has already been shown that early sexual maturation and high fertility of females, which can also be considered a peak physical performance, come at a longevity cost [4-6]. Especially under adverse environmental conditions, the average lifespan of fertile women is reduced by fatal consequences of pregnancy and childbirth. Moreover, experimental models and observations in humans also provide evidence that increases in fecundity come with a longevity cost [7, 8, 20, 21].

Whereas previous studies on the cost of sexual development and success could only reliably be estimated in women, we here show for the first time that early and extraordinary peaks in physical performance trades off at longevity in men. Physical excellence and sports may have a direct cost due to intense training and fierce competition, especially when there is a high risk of bodily collision or levels of physical contact [16]. It is less likely that these direct costs explain the observed trade-off between early and extraordinary physical performance and longevity, since we showed a similar trade off when analyzing residual life expectancy from age 50. This finding supports the idea that early and extraordinary peak performance comes with a higher pace of ageing [22, 23]. It is tempting to speculate about the underlying biological mechanisms of this developmental constraint. Some have suggested that growth and subsequently, larger size, result in a body which costs more energy to maintain, explaining the higher pace of ageing [24, 25]. The mTOR pathway, which regulates growth in early life and pace of ageing in late life, is a potential molecular pathway that can explain for the observed trade off [26-28]. Others have suggested that hormonal regulation of development and maintenance could play a role, as has been observed for the GH-IGF signaling pathway explaining the size-life span trade off in domestic dogs, and the muscle mass-immune competence trade-off mediated by testosterone observed in primates and other species [10, 29-32]. All mechanistic explanations are plausible and it needs to be studied which pathways are causal, and at which we can intervene to secure longer and healthier lives.

## METHODS

### Experimental design

This study is an observational cohort study to investigate the effect of the age at, and relative rank of personal record on age at death in Olympic track and field athletes.

### Study population

Data on year of birth, year of death, discipline, year of personal record, performance score of personal record and nationality were retrieved from the continuously updated and most comprehensive online database on Olympic athletes: Sports Reference database, in April 2014 [13]. We used this database previously to determine the difference in mortality between disciplines with different cardiovascular intensity [16]. We included all Olympic track and field athletes with a known personal record. This group consisted of 3671 athletes. First, we excluded 2274 athletes who were born after 1913 to ensure that we had a cohort of individuals with a complete life history. Second, we excluded 126 athletes with an unknown date of birth and 216 athletes with an unknown age of death. In an additional analysis, we excluded athletes who died before age 50 (N=120), to minimize the effect of survival bias and extrinsic or accidental causes of mortality. For one of the analyses, we excluded all personal records set after the year 1935, effectively before the effective doping became available. Recording of personal records was not bound to the Olympic Games and also include every official competition, excluding trainings.

### Disciplines

The track and field category consisted of 58 disciplines. In the throwing and jumping disciplines we used distance as a measure, in the running disciplines we converted time over a fixed distance to average speed for reasons of comparison, and in the pentathlon and decathlon we used the number of points. Variation in the number of points in the pentathlon and decathlon was high enough to be a good reflection of the athlete's performance.

The performance score of personal record was average speed (m s-1) for the running or walking disciplines, distance (m) for the throwing and jumping disciplines or number of points for the pentathlon and decathlon.

When categorising the various disciplines per cardiovascular intensity category we used the classification of sports published in the Journal of American college of cardiology, as we have used before [15, 16]. In the analysis where we grouped multiple disciplines according to the International Association of Athletics Federation (IAAF) classification before stan-dardization, we categorized the disciplines in a short, middle, long, jumping and throwing category [14].

### Statistical analysis

For means of comparison between disciplines we standardized age and performance score of the personal records. We divided the deviation from the mean age at personal record of the discipline and sex for each discipline and sex separately. The same was done for the performance score of the personal record to derive the relative rank of the personal record. Additionally, we grouped personal records per cardiovascular intensity category or per IAAF category, as described earlier, and standardized age and performance score in these groups to derive an alternative method for estimating the relative age and rank of the personal record.

Most athletes had more than one personal record and had therefore more than one age and performance score of personal record. For the primary analysis we calculated the average age and rank of the personal record for each of the disciplines, all after standardising. As an additional analysis we used the best personal record of an athlete, their primary discipline, using the record with the highest rank.

We used a left truncated cox proportional hazard model to calculate hazard ratios for mortality from the age of the peak performance forward. We used estimated marginal means from a linear mixed model to estimate the mean age at death in groups. The linear mixed model was applied on the subset of 844 male athletes who died after age 50, to artificially left truncate the linear mixed model. All estimates were adjusted for sex, year of birth and nationality where possible.

All analyses were performed using IBM SPSS Statistics 22.0 (IBM Corp., Armonk, NY).

## SUPPLEMENTARY DATA TABLE



## References

[1] Harvey PH, Zammuto RM (1985). Patterns of mortality and age at first reproduction in natural populations of mammals. Nature.

[2] Ingram DK, Reynolds MA, Les EP (1982). The relationship of genotype, sex, body weight, and growth parameters to lifespan in inbred and hybrid mice. Mech Ageing Dev.

[3] Rollo CD (2002). Growth negatively impacts the life span of mammals. Evol Dev.

[4] Jacobsen BK, Oda K, Knutsen SF, Fraser GE (2009). Age at menarche, total mortality and mortality from ischaemic heart disease and stroke: the Adventist Health Study, 1976-88. Int J Epidemiol.

[5] Jacobsen BK, Heuch I, Kvåle G (2007). Association of low age at menarche with increased all-cause mortality: a 37-year follow-up of 61,319 Norwegian women. Am J Epidemiol.

[6] Lakshman R, Forouhi NG, Sharp SJ, Luben R, Bingham SA, Khaw K-T, Wareham NJ, Ong KK (2009). Early age at menarche associated with cardiovascular disease and mortality. J Clin Endocrinol Metab.

[7] Westendorp RG, Kirkwood TB (1998). Human longevity at the cost of reproductive success. Nature.

[8] Wang X, Byars SG, Stearns SC (2013). Genetic links between post-reproductive lifespan and family size in Framingham. Evol Med Public Health.

[9] Fink B, Neave N, Seydel H (2007). Male facial appearance signals physical strength to women. Am J Hum Biol.

[10] Muehlenbein MP, Bribiescas RG (2005). Testosterone-mediated immune functions and male life histories. Am J Hum Biol..

[11] Donato AJ, Tench K, Glueck DH, Seals DR, Eskurza I, Tanaka H (2003). Declines in physiological functional capacity with age: a longitudinal study in peak swimming performance. J Appl Physiol (1985).

[12] Ericsson KA, Baltes PB, Baltes MM (1993). Peak performance and age: an examination of peak performance in sports. Successful aging: Perspectives from the behavioral sciences.

[13] Sports Reference LL. (2014). Olympics at Sports-Reference.com - Olympic Statistics and History. http://www.sports-reference.com/olympics.

[14] International Association of Athletics Federation (2016). IAAF: Disciplines. http://www.iaaf.org/disciplines.

[15] Mitchell JH, Haskell W, Snell P, Van Camp SP (2005). Task Force 8: classification of sports. J Am Coll Cardiol..

[16] Zwiers R, Zantvoord FW, Engelaer FM, van Bodegom D, van der Ouderaa FJ, Westendorp RG (2012). Mortality in former Olympic athletes: retrospective cohort analysis. BMJ.

[17] Holt RI, Erotokritou-Mulligan I, Sönksen PH (2009). The history of doping and growth hormone abuse in sport. Growth Horm IGF Res..

[18] Yesalis CE, Bahrke MS History of doping in sport. Performance enhancing substances in sport and exercise Champaign. Human Kinetics.

[19] Promislow DE, Harvey PH (1990). Living fast and dying young: A comparative analysis of life‐history variation among mammals. J Zool (Lond).

[20] Lycett JE, Dunbar RI, Voland E (2000). Longevity and the costs of reproduction in a historical human population. Proc Biol Sci.

[21] Meeûs D (2000). Human longevity at the cost of reproductive success: evidence from global data. J Evol Biol.

[22] Baudisch A (2011). The pace and shape of ageing. Methods Ecol Evol..

[23] Finch CE (1994). Longevity, senescence, and the genome.

[24] Wensink MJ, van Heemst D, Rozing MP, Westendorp RG (2012). The maintenance gap: a new theoretical perspective on the evolution of aging. Biogerontolo-gy.

[25] Blagosklonny MV (2013). Big mice die young but large animals live longer. Aging (Albany NY).

[26] Leontieva OV, Paszkiewicz GM, Blagosklonny MV (2012). Mechanistic or mammalian target of rapamycin (mTOR) may determine robustness in young male mice at the cost of accelerated aging. Aging (Albany NY).

[27] Blagosklonny MV (2010). Why men age faster but reproduce longer than women: mTOR and evolutionary perspectives. Aging (Albany NY).

[28] Blagosklonny MV, Hall MN (2009). Growth and aging: a common molecular mechanism. Aging (Albany NY).

[29] Berryman DE, Christiansen JS, Johannsson G, Thorner MO, Kopchick JJ (2008). Role of the GH/IGF-1 axis in lifespan and healthspan: lessons from animal models. Growth Horm IGF Res.

[30] Greer KA, Canterberry SC, Murphy KE (2007). Statistical analysis regarding the effects of height and weight on life span of the domestic dog. Res Vet Sci.

[31] Kraus C, Pavard S, Promislow DE (2013). The size-life span trade-off decomposed: why large dogs die young. Am Nat.

[32] Sutter NB, Bustamante CD, Chase K, Gray MM, Zhao K, Zhu L, Padhukasahasram B, Karlins E, Davis S, Jones PG, Quignon P, Johnson GS, Parker HG (2007). A single IGF1 allele is a major determinant of small size in dogs. Science.

