# Supplementary material for: Early and extraordinary peaks in physical performance come with a longevity cost
Source: Aging (Albany NY). 2016 Aug 19;8(8):1822–8. doi: 10.18632/aging.101023 (PMC5032698; doi:10.18632/aging.101023)
Supplement: Supplementary file 1 [file aging-08-1822-s001.pdf]

SUPPLEMENTARY DATA

Table S1. Characteristics of included athletes

Athletes (n)	1055
Male (n)	958
Female (n)	97
Year of birth (median, range)	1897 (1864-1913)
Year of death (median, range)	1969 (1901-2010)
Age at death (mean, SD)	72.1 (16.9)
Personal records (n)	2320
Personal records per athlete (median, range)	2 (1-10)
Age at personal record (mean, SD)	24.9 (3.8)
Disciplines (n)	58
Nationalities (n)	41

SD=Standard deviation
